# Supplementary material for: Predicting the effects of deep brain stimulation using a reduced coupled oscillator model
Source: PLoS Comput Biol. 2019 Aug 8;15(8):e1006575. doi: 10.1371/journal.pcbi.1006575 (PMC6701819; doi:10.1371/journal.pcbi.1006575)
Supplement: S1 Appendix — This appendix demonstrates a particular property of Hilbert transform, which is used in the main text to show that there exists a direct relationship between the neural activity in the model and the signal in the experiment. (PDF) [file pcbi.1006575.s001.pdf]

## S1 Relating the Order Parameter to the Analytic Signal

In the main text we show that there exists a direct relationship between the neural activity (which we model) and the signal we measure in experiment if the following is satisfied for a given oscillator $\theta_n(t)$

$$\hat{H}[\cos(\theta_n)] = \sin(\theta_n), \tag{1}$$

where $\hat{H}$ denotes the Hilbert transform. Here we establish under what conditions Eq (1) is satisfied exactly and we show numerically that the identified condition is indeed typically satisfied.

For a function $\theta_n(t)$ describing changes in phase of the oscillator on interval $t \in [0, T]$, let us define a complex valued function $A(t) = e^{i\theta_n(t)}$, and expand it as a periodic function with period $T$ into a Fourier series

$$A(t) = \sum_{n=-\infty}^{\infty} C_n e^{i\omega_n t}, \tag{2}$$

with

$$\omega_n = \frac{n2\pi}{T}. \tag{3}$$

**Lemma 1.** *If $A(t)$ has no power in the negative frequencies, i.e., $C_n = 0$ for $n < 0$, then $\hat{H}[\cos(\theta_n)] = \sin(\theta_n)$.*

*Proof.* Using the Euler relation

$$A(t) = \sum_{n=-\infty}^{\infty} C_n \cos(\omega_n t) + i \sum_{n=-\infty}^{\infty} C_n \sin(\omega_n t). \tag{4}$$

Splitting up into positive and negative frequencies

$$A(t) = C_0 + \sum_{n=-\infty}^{-1} C_n \cos(\omega_n t) + \sum_{n=1}^{\infty} C_n \cos(\omega_n t) + i \sum_{n=-\infty}^{-1} C_n \sin(\omega_n t) + i \sum_{n=1}^{\infty} C_n \sin(\omega_n t). \tag{5}$$

Which can be written as

$$A(t) = C_0 + \sum_{n=1}^{\infty} (C_n + C_{-n}) \cos(\omega_n t) + i \sum_{n=1}^{\infty} (C_n - C_{-n}) \sin(\omega_n t). \tag{6}$$

We now consider $A(t)$ to be a function with no power in the negative frequencies, i.e. the Fourier coefficients corresponding to the negative frequencies are zero, leading to

$$A(t) = \sum_{n=0}^{\infty} C_n \cos(\omega_n t) + i \sum_{n=0}^{\infty} C_n \sin(\omega_n t). \tag{7}$$

Expressing $C_n$ in terms of the real Fourier coefficients $a_n$ and $b_n$, i.e. $C_n = a_n - ib_n$ leads to

$$A(t) = \sum_{n=0}^{\infty} [a_n \cos(\omega_n t) + b_n \sin(\omega_n t)] + i \sum_{n=0}^{\infty} [a_n \sin(\omega_n t) - b_n \cos(\omega_n t)]. \tag{8}$$

Using the fact that $\hat{H}[\cos(\omega_n t)] = \sin(\omega_n t)$ and $\hat{H}[\sin(\omega_n t)] = -\cos(\omega_n t)$,

$$A(t) = \sum_{n=0}^{\infty}[a_n \cos(\omega_n t) + b_n \sin(\omega_n t)] + i\hat{H}\sum_{n=0}^{\infty}[a_n \cos(\omega_n t) + b_n \sin(\omega_n t)]. \tag{9}$$

Or

$$A(t) = g(t) + i\hat{H}[g(t)], \tag{10}$$

where $g(t)$ is some real valued time series of length $T$. This shows that if some complex valued time series $A(t)$ has no power in the negative frequencies, then it is always possible to express it in the form given by Eq. (10). Using this result, we can say that if $e^{i\theta_n}$ has no power in the negative frequencies, then it must be possible to write

$$e^{i\theta_n} = g(t) + i\hat{H}[g(t)]. \tag{11}$$

Using the Euler relation,

$$e^{i\theta_n} = \cos(\theta_n) + i\sin(\theta_n). \tag{12}$$

Comparing real and imaginary parts,

$$g(t) = \cos(\theta_n), \tag{13}$$

and

$$\hat{H}[g(t)] = \sin(\theta_n). \tag{14}$$

□

We now demonstrate that if $\theta_n$ evolves according to the Kuramoto equations (with a noise term), then $e^{\theta_n}$ typically has negligible power in the negative frequencies. We would also like to point out that if $\frac{d\theta_n}{dt} = \omega_n$ then $\theta_n(t) = \omega_n t$ and $e^{i\theta_n} = \cos(\omega_n t) + i\sin(\omega_n t)$. From the definition of the Fourier transform, we can see that $e^{i\theta_n}$ has only a single frequency component, which is $\omega_n$. If $\omega_n > 0$, then $e^{i\theta_n}$ would indeed have no power in the negative frequencies and we can see that Eq (1) is indeed satisfied.

The Kuramoto equations with an additional noise term for $\theta_n$ are given by

$$d\theta_n = [\tilde{\omega}_n + k\rho\sin(\psi - \theta_n)]dt + \sigma\sqrt{dt}N(0,1), \tag{15}$$

where $\tilde{\omega}_n$ is the natural frequency of oscillation, $k$ is the coupling constant, $\sigma$ is the noise strength and $N(0,1)$ denotes a number drawn from a standard normal distribution. The time evolution for $\theta_n$ can be seen to consist of a linear and nonlinear part, with the nonlinear part describing the effects of coupling and noise. We generally expect the linear part to be strongly positive, with the effects of coupling and noise being relatively small. For such a system, the evolution for each $\theta_n$ is expected to be approximately monotonic and increasing, thus $e^{i\theta_n(t)}$ is expected to have a negligible negative frequency component. To illustrate this, we used 1000 trials of simulated data, with each trial producing a time series of oscillation data generated according to the Kuramoto model with a randomly generated set of parameters. In each case the parameters were sampled from a uniform distribution with ranges chosen to produce data that resembles the tremor oscillations found in ET patients. We know, for example, that tremor oscillations are typically around 5 Hz, so we choose a range for $\omega_0$ which captures this. An example of the oscillation data generated can be seen in Figure 1. The natural frequencies $\{\tilde{\omega}_n\}$ were sampled from a normal distribution with mean $\omega_0$ and standard deviation $\gamma$. The ranges for each parameter are given in Table 1. For each trial, we calculate the fraction of power in the negative frequencies averaged across oscillators. The results shown in Figure 2 show that, in the majority of cases, the fraction of power in the negative frequencies averaged across oscillators is negligible.

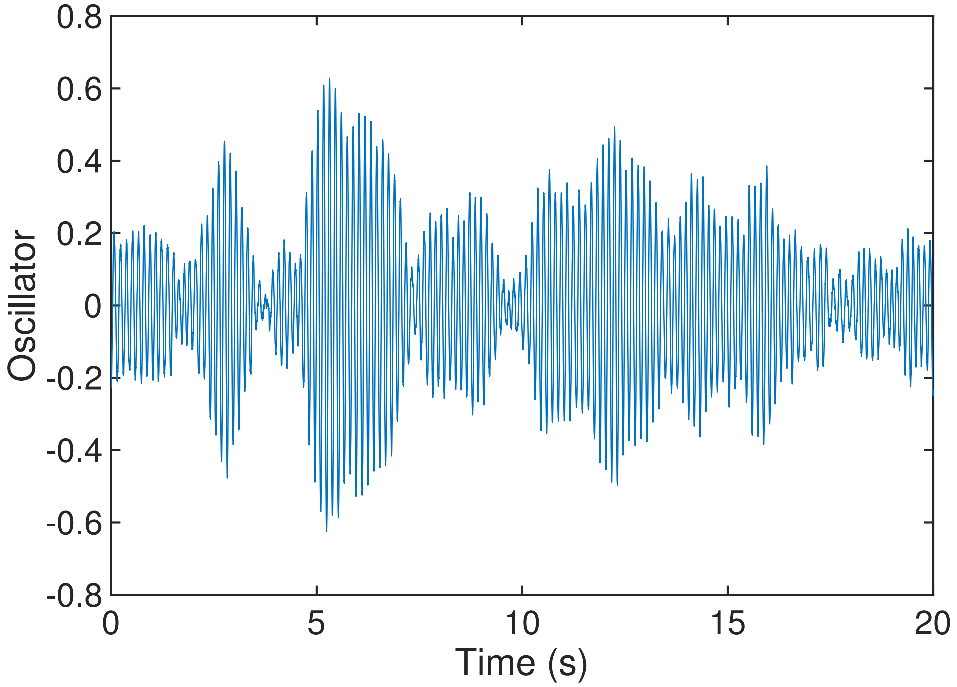


**Figure 1.** A single trial of data output from simulating the Kuramoto equations. We choose the parameter ranges so as to closely resemble tremor data.

| Parameter | Min | Max |
|---|---|---|
| $k$ | 1 | 10 |
| $\omega_0$ | 4 | 12 |
| $\gamma$ | 0 | 1 |
| $\sigma$ | 1 | 5 |

**Table 1.** Minimum and maximum bounds when sampling from a uniform distribution for each parameter used to simulate the Kuramoto equations. Note that the units of $\omega_0$ and $\gamma$ are Hz.

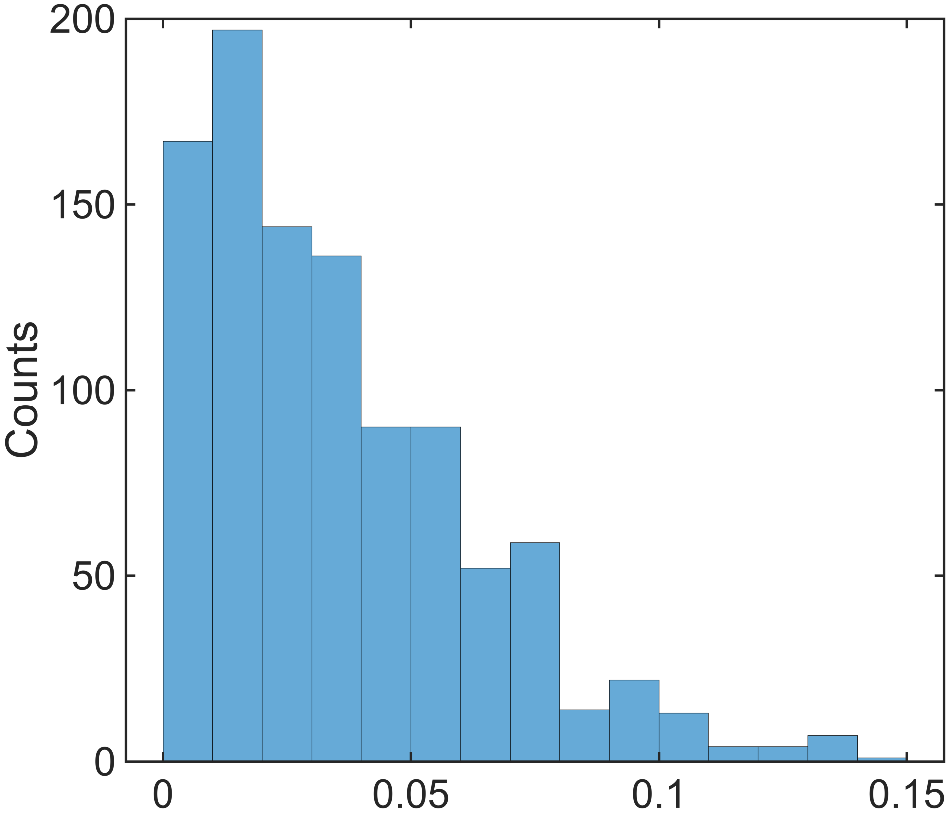

**Figure 2.** Plot showing the frequency of trials against fraction of power in the negative frequencies averaged over oscillators.
